# Implementation of a Clinical Reasoning Course in the Internal Medicine trimester of the final year of undergraduate medical training and its effect on students' case presentation and differential diagnostic skills

**DOI:** 10.3205/zma001143

**Published:** 2017-11-15

**Authors:** Sigrid Harendza, Ingo Krenz, Andreas Klinge, Ulrike Wendt, Matthias Janneck

**Affiliations:** 1 University Medical Centre Hamburg-Eppendorf, III. Department of Internal Medicine, Hamburg, Germany; 2 Praxis Blankenese, Consultant in Internal Medicine/Nephrology/Hypertensiology, Hamburg, Germany; 3 Diabetes Schwerpunktpraxis Eidelstedt, Consultant in Internal Medicine/ Diabetology, Hamburg, Germany; 4 Bethesda Hospital Bergedorf, Department of Psychiatry and Psychotherapy, Hamburg, Germany

**Keywords:** analytical thinking, case presentation, clinical reasoning, cognitive errors, differential diagnosis, internal medicine, intuitive thinking, uncertainty

## Abstract

**Background:** Clinical reasoning, comprising the processes of clinical thinking, which form the basis of medical decisions, constitutes a central competence in the clinical routine on which diagnostic and therapeutic steps are based. In medical curricula in Germany, clinical reasoning is currently taught explicitly only to a small extend. Therefore, the aim of this project was to develop and implement a clinical reasoning course in the final year of undergraduate medical training.

**Project description: **A clinical reasoning course with six learning units and 18 learning objectives was developed, which was taught by two to four instructors on the basis of 32 paper cases from the clinical practice of the instructors. In the years 2011 to 2013, the course of eight weeks with two hours per week was taught seven times. Before the first and after the last seminar, the participating students filled out a self-assessment questionnaire with a 6-point Likert scale regarding eight different clinical reasoning skills. At the same times, they received a patient case with the assignment to prepare a case presentation and differential diagnoses.

**Results: **From 128 participating students altogether, 42 complete data sets were available. After the course, participants assessed themselves significantly better than before the course in all eight clinical reasoning skills, for example in “Summarizing and presentation of a paper case” or in the “Skill to enumerate differential diagnoses” (p<0.05). The greatest increase occurred in the skill to recognize typical cognitive errors in medicine and to identify risk situations for their occurrence (pre: 2.98±0.92 and retro-pre: 2.64±1.01, respectively, versus post: 4.38±0.88). Based on the ratio of number of words used per keywords used the problem presentation of the paper case was significantly more focused after the course (p=0.011). A significant increase in the number of gathered differential diagnoses was not detected after the course.

**Conclusion: **The newly developed and established Clinical Reasoning Course leads to a gain in the desired skills from the students’ self-assessment perspective and to a more structured case presentation. To establish better options to exercise clinical reasoning, a longitudinal implementation in the medical curriculum seems to be desirable. Faculty training would be useful to implement the concept as standardized as possible.

## Zusammenfassung

**Hintergrund: **Clinical Reasoning, also klinische Denkprozesse, die medizinischen Entscheidungen zugrunde liegen, stellt im ärztlichen Alltag eine zentrale Kompetenz dar, auf der die Diagnostik- und Behandlungsschritte basieren. In medizinischen Curricula in Deutschland wird Clinical Reasoning bisher nur wenig explizit unterrichtet. Ziel dieses Projektes war daher die Entwicklung und Implementierung eines Clinical-Reasoning-Kurses im Praktischen Jahr.

**Projektbeschreibung:** Es wurde ein Clinical-Reasoning-Kurs mit sechs Lerneinheiten und 18 Lernzielen konzipiert, der von zwei bis vier Dozierenden auf der Basis von 32 Patientenfällen aus dem Arbeitsalltag der Dozierenden unterrichtet wurde. In den Jahren 2011 bis 2013 fand der Kurs zu je acht Doppelstunden im PJ-Tertial Innere Medizin insgesamt siebenmal statt. Die teilnehmenden Studierenden füllten vor dem ersten und nach dem letzten Kurstermin einen Selbsteinschätzungsbogen mit einer 6-stufigen Likert-Skala zu acht verschiedenen Clinical-Reasoning-Fähigkeiten aus. Zu denselben Zeitpunkten erhielten sie einen Patientenfall, der im Hinblick auf Problempräsentation und Differentialdiagnosen zu bearbeiten war.

**Ergebnisse:** Bei insgesamt 128 teilnehmenden Studierenden waren 42 komplette Datensätze verfügbar. Nach dem Kurs schätzten sich die Teilnehmenden in allen acht Clinical-Reasoning-Fähigkeiten, beispielsweise in der „Zusammenfassung und Präsentation eines Patientenfalls“ oder in der „Fähigkeit, Differentialdiagnosen zu benennen“, signifikant besser ein als vor dem Kurs (p<0,05). Der größte Zuwachs zeigte sich bei der Fähigkeit, typische ärztliche Denkfehler zu erkennen und Risikosituationen für deren Entstehung zu identifizieren (pre: 2,98±0,92 bzw. retro-pre: 2,64±1,01 versus post: 4,38±0,88). Gemessen an der Zahl der im Verhältnis zur Nennung von Schlüsselbegriffen verwendeten Wörter war die Problempräsentation des Patientenfalls nach dem Kurs signifikant fokussierter (p=0,011). Eine signifikante Steigerung der Anzahl von erarbeiteten Differentialdiagnosen fand sich nach dem Kurs nicht.

**Schlussfolgerung: **Der neu konzipierte und etablierte Clinical-Reasoning-Kurs führt in der Selbsteinschätzung der Studierenden zu einem Zuwachs der gewünschten Fähigkeiten und zu einer strukturierteren Fallpräsentation. Um noch bessere Übungsmöglichkeiten für Clinical Reasoning zu schaffen, scheint eine longitudinale Implementierung in das medizinische Curriculum wünschenswert. Eine Trainingsmaßnahme für Dozierende wäre sinnvoll, damit das Konzept möglichst standardisiert umgesetzt werden kann.

## Introduction

In the interaction with patients, physicians make dozens of decisions every day. The term clinical reasoning refers to the thinking processes, which form the basis for these decisions. These include “medical problem solving” and “medical decision making” [1] and “diagnostic reasoning” and “therapeutic reasoning”, respectively [2]. A sequence of different steps can be described for the process of medical problem solving: to collect information, to analyse information and to name differential diagnoses, to collect more information, to gain an inner representation of the case, to weigh and review the differential diagnoses and to decide about further steps [3]. In this process, the hypothetic-deductive process and the recognition of illness scripts are different approaches, which are connected with different levels of expertise [4], [5].

Kahneman identified an intuitive and an analytical form for decision making in general [6]. For medical decisions, both thinking processes, the intuitive-unconscious and the analytical-conscious type, have been described [7]. However, especially the intuitive way of clinical thinking is very prone to typical cognitive errors [8]. Since especially medical students and young residents easily succumb to heuristic errors [9] that can lead to malpractice, teaching clinical reasoning including possible cognitive errors seems to be an essential goal, which contributes to patient safety.

The international literature provides good tips and instructions how to teach clinical reasoning [10], [11]. Schmidt and Mamede particularly argue based on empirical research that medical students benefit from different types of clinical reasoning teaching in the different phases of their undergraduate medical education [12]. In 2014, a support program for medical students and physicians was established in the USA to eliminate deficits in clinical reasoning skills [13]. In the German language area, clinical reasoning is only sporadically taught explicitly [14], [http://www.klinikum.uni-muenchen.de/Institut-fuer-Didaktik-und-Ausbildungsforschung-in-der-Medizin/de/forschung/projekte_aktuell/ccd.html, accessed 14.4.2017]. Thus, in this project, a course concept for clinical reasoning for undergraduate medical students in their final year was developed based on the described theoretical framework and tested at the Medical Faculty of Hamburg University.

## Project Description

Within the Internal Medicine trimester, at total of 16 two-hour seminars are scheduled for final year undergraduate medical students at the University Medical Center Hamburg-Eppendorf. Of these, eight are taught by the III. Department of Internal Medicine. Per seminar, usually two final year students present a patient case from their ward that is subsequently discussed together with the instructors.

**Figure 1:**
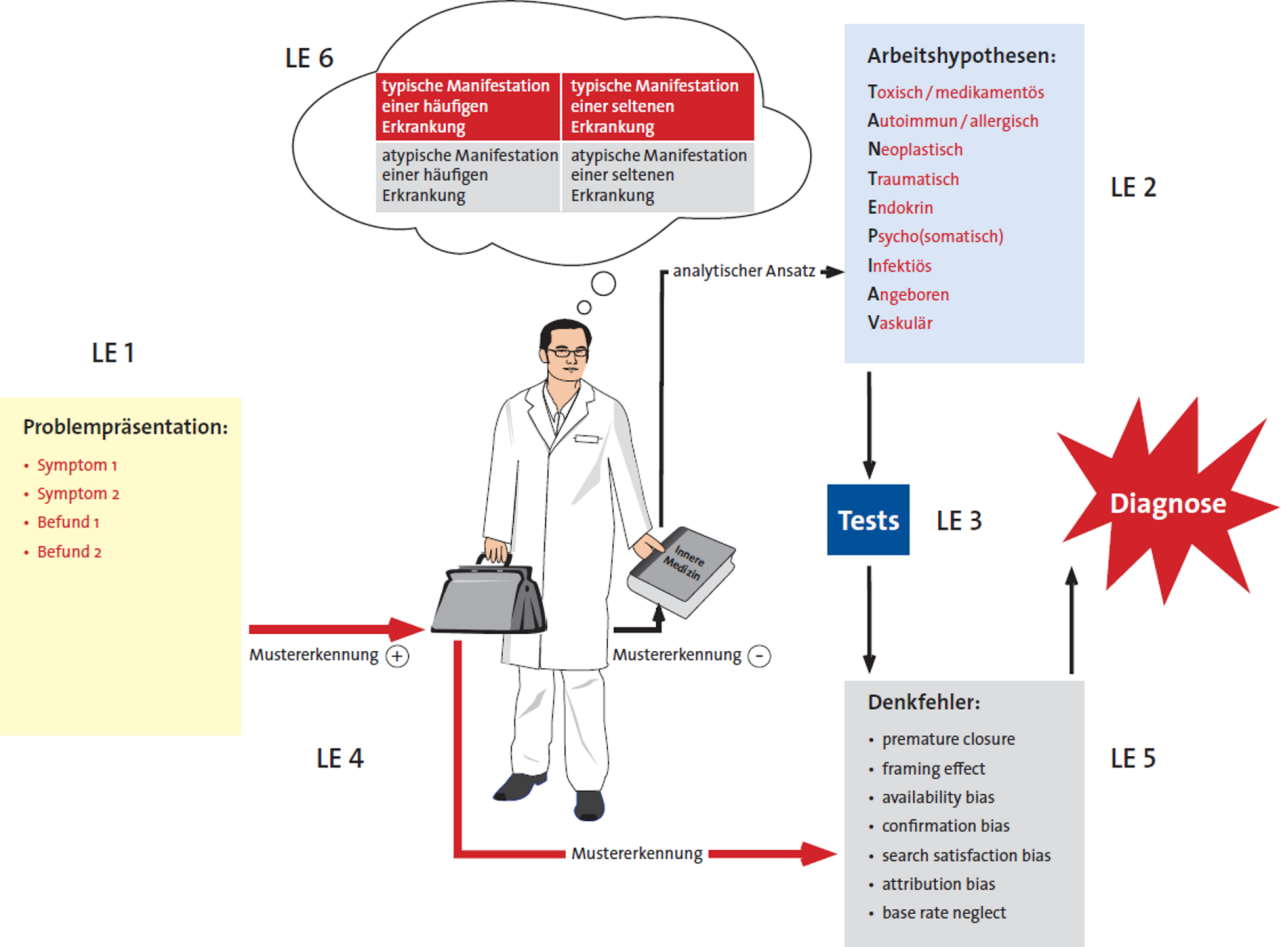
Course concept Clinical Reasoning with learning units (LU)

**Figure 2:**
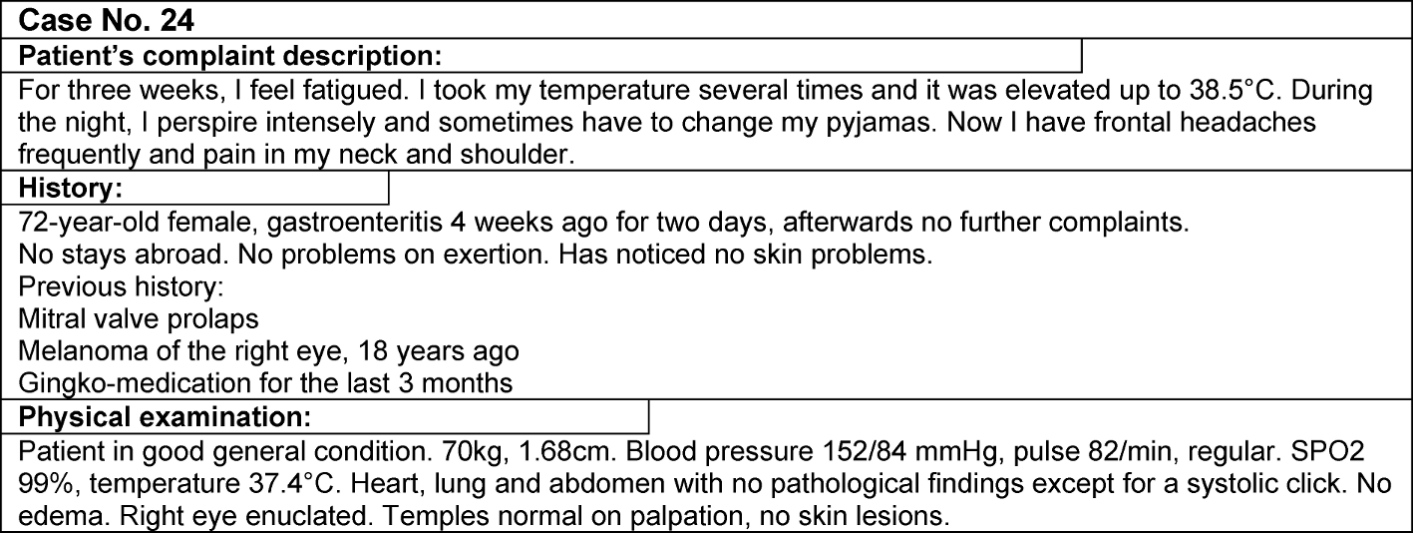
Format of the paper cases (example)

The eight seminars taught by the III. Department of Internal Medicine were transformed for the newly developed clinical reasoning course concept. In the first step, based on the steps of the clinical reasoning process [11], six learning units (LU) were defined, which illustrate the outline of the course (see Figure 1 (Fig. 1)): “data acquisition, correct/abstract presentation of the problem” (LU 1), “deductive reasoning of differential diagnoses” (LU 2), “diagnoses on the test rig and theory of testing” (LU 3), “pattern recognition” (LU 4), “cognitive errors” (LU 5), and “dealing with uncertainty” (LU 6). Corresponding learning objectives were assigned to these learning units (see Table 1 (Tab. 1)). For the different learning objectives, SH, IK, AK, and MJ collected 32 suitable patient cases from their clinical practise, which were edited in a standardized and useful manner for course purposes (see Figure 2 (Fig. 2)). Only real patient cases from the authors’ own experience were used for all learning units including the different cognitive errors to organize the course in a lively way and to talk, e.g. about how to deal with one’s own uncertainty, in a very authentic manner. The thinking processes, including the erroneous ones, were made transparent and discussed. The eight seminars were conducted by two to four instructors (SH, IK, AK, MJ) according to the sandwich principle [15]. After a short discussion of an ongoing patient case matching the respective learning unit as an introduction, the students worked in small groups with different paper cases, followed by a short lecture by the instructors with content matching the respective topic of the learning unit, and by further processing and discussion of the paper cases with the whole group. Either the short lecture consisted of a dialog between the instructors about a certain problem, e.g. how one manages to endure uncertainty, or it included prepared slides with visual diagnoses to practise pattern recognition or computational examples to estimate the quality of tests. The instructors’ strengths for the different topics were used accordingly, again to obtain the most authentic teaching, especially for the topic “cognitive errors” and “dealing with uncertainty”. It was also important that the cases were not solved during the seminars but that the solutions were developed during the course or partly remained unsolved until the end of the course.

To assess the learning success, a self-assessment questionnaire was developed, which included the learning objectives of the course summarized in eight main skill that play a role in clinical reasoning. The extent of each respective skill had to be specified on a 6-point Likert scale (1: does not apply, 6: does apply very much). This questionnaire had to be completed by the participating students before the first seminar (pre) and after the last seminar (post). After the last seminar, the students were also asked to assess their clinical reasoning skills before the course retrospectively (retro-pre). The Cronbach’s alpha values were 0.80 (pre), 0,91 (retro-pre), and 0,85 (post). Furthermore, the students received the same patient case (see Figure 2 (Fig. 2)) at the beginning of the first seminar and at the end of the last seminar and were asked to write a problem presentation for this case and to make a list of weighted differential diagnoses.

Since February 2011, this course of eight weeks is offered. Seven complete courses are included in this study (until February 2013). Finial year students in their internal medicine trimester at University Medical Center Hamburg-Eppendorf participate voluntarily. Students were only included in the statistical evaluation, who participated in at least five of the eight seminars und who completed the self-assessment questionnaire and the paper case assessment before and after the course. The program SPSS/PAWS 18 was used for statistical evaluation. Means (MW) and standard deviations (SD) are presented which showed significant differences (p<0.05) calculated with the Wilcoxon signed-rank test.

## Results

In the six cycles of the course, 128 students participated altogether. However, only 42 students (31 women, 11 men) could be included who fulfilled the evaluation criteria.

With respect to their skill in the clinical reasoning, process which resembled the learning objectives of the course, the students assessed themselves significantly better in all eight skills after the course (post) than before the course (pre as well as retro-pre) (see Table 2 (Tab. 2)). The greatest increment appeared in the skill to assess typical reasoning errors and to identify situations when they can occur. For the skill to recognize uncertainty in medical decisions and to communicate it to a patient and for the skill to assess test results with respect to their relevance for a specific case, students assessed themselves retro-pre to be significantly less skilled compared to their pre assessment. The latter skill shows the greatest difference between retro-pre and pre overall.

The results of the students’ workup of the paper case before and after the course are displayed in table 3 (Tab. 3). The number of words for the case presentation sank significantly for the workup of the paper case after the course. This was accompanied by a significantly better ratio of keywords minus mistakes per total word count. The number of mistakes was lower for the workup of the paper case after the course and the number of differential diagnoses was higher, yet these changes were not significant.

## Discussion

The participating students assessed themselves to be significantly more skilled in all eight clinical reasoning skill than before the course, in comparison of the pre and post assessment as well as in the retro-pre and post assessment. The skill to summarize a complex patient case in 2-3 sentences received the highest marks in the self-assessment before as well as after the course. Furthermore, the problem presentation was significantly more focused in the summary of the patient case after the course than before the course. The teaching format how to present a problem seems to be quite suitable in this course to learn problem presentation. However, a sound case presentation can also be learned well by video-based instruction [16]. That the students had assessed themselves before the course too well in the skill to assess test results with respect to their relevance for a specific case is demonstrated by their significantly lower assessment for this skill after the course. Apparently, the skill to assess test results with respect to their relevance for a specific case is practiced not enough in undergraduate medical education in Germany. This is demonstrated in another study, where German medical students order significantly more laboratory and radiology tests in a study with simulated patients compared with Dutch medicals students, while there is no difference in making the correct diagnosis between the two groups [17]. The learning progress experienced by the students with respect to ordering medical tests seems to be a reference that the awareness for the relevance of overdiagnosis can be raised by a case based clinical reasoning course in a way similar to the results presented for a problem based course for incorrect prescriptions of medication [18].

In the retro-pre assessment, the item for pattern recognition, namely to immediately assign a diagnosis to typical patterns of a specific case, showed the highest mean and the smallest difference compared to the pre assessment. This might be due to the fact, that pattern recognition is trained implicitly during undergraduate medical education in multiple choice exams that reach beyond mere knowledge by including short case presentations [19]. The training of pattern recognition in the clinical reasoning course leading to a significantly increased self-assessment in this skill speaks in the favor of additional explicit learning of pattern recognition. For the skill to correctly interpret an ECG it has been demonstrated that the combination of an implicit and an explicit learning method lead to the best results [20].

To switch from intuitive to analytical thing, as it was taught in the clinical reasoning course, seems to be an important medial skill to make correct diagnoses when internal medicine patient cases are complex [21]. The most important diagnostic errors in clinical reasoning [8] were discussed in our course in a metacognitive way and substantiated with examples from everyday life. The skill to recognize typical reasoning errors and to identify situations when they can occur showed the greatest increase in students’ self-assessment after the course. Hence, it seems to be relevant to make cognitive errors explicit to reveal the problem of cognitive errors. However, exclusive teaching strategies how to recognize cognitive errors are not effective in reducing cognitive errors [22]. A better organization of medical knowledge showed a small, but consistent benefit for the reduction of the occurrence of cognitive errors [23].

To broach the issue of dealing with uncertainty in the clinical reasoning course seems to be an important learning objective, because dealing with uncertainty is part of the daily medical activity and the results of a survey among medical students shows that their self-perception is characterized by feelings of powerlessness and uncertainty [24]. Clinical reasoning needs to be learned and assessed in the context of uncertainty and with respect to the awareness that – contrary to multiple choice based assessments – more than one answer can be correct in this process. This is displayed in a framework by Cooke and Lemay, which includes increasing levels of difficulty of clinical reasoning skills [25]. That would qualify clinical reasoning skills to be embedded longitudinally into the undergraduate medical curriculum.

A strength of this project is the implementation of a clinical reasoning course based on a framework with defined learning objectives, matching teaching and learning methods and an assessment of the learned skills in the sense of constructive alignment [26]. This permitted the assessment of some clinical reasoning skills before and after the course. A weakness of this project is the self-assessment of their clinical reasoning skills by the students because self-assessment of students with respect to skills correlates often only to a small extent with their real skills [27]. With the additional use of a retro-pre survey, a correct self-assessment can be assumed though [28]. Another weakness of this project is implementation in different turns of the final year and the high number of course participants whose data could not be included in the study due to missing documents. Despite these limitations, the results of this project are good indicators for the usefulness and effectiveness of this newly established clinical reasoning course. 

## Conclusion

The clinical reasoning course, which was designed for and implemented in the internal medicine trimester of the final year curriculum at Hamburg Medical Faculty leads to self-perceived increase in clinical reasoning skills by the participating students and to a more structured case presentation. This concept could also be developed at other medical schools whereat the authenticity of the instructors and the use of internal paper cases seem to be of crucial importance. To generate explicit options to exercise clinical reasoning skills, a longitudinal implementation of clinical reasoning in the curriculum seems to be worthwhile. It should be accompanied by faculty training to establish the course concept in the desired way and as standardized as possible.

## Acknowledgements

We thank all medical students who participated in this newly established clinical reasoning course.

## Competing interests

The authors declare that they have no conflict of interest in connection with this article. Parts of this article originate from UW’s doctoral thesis.

## Figures and Tables

**Table 1:**
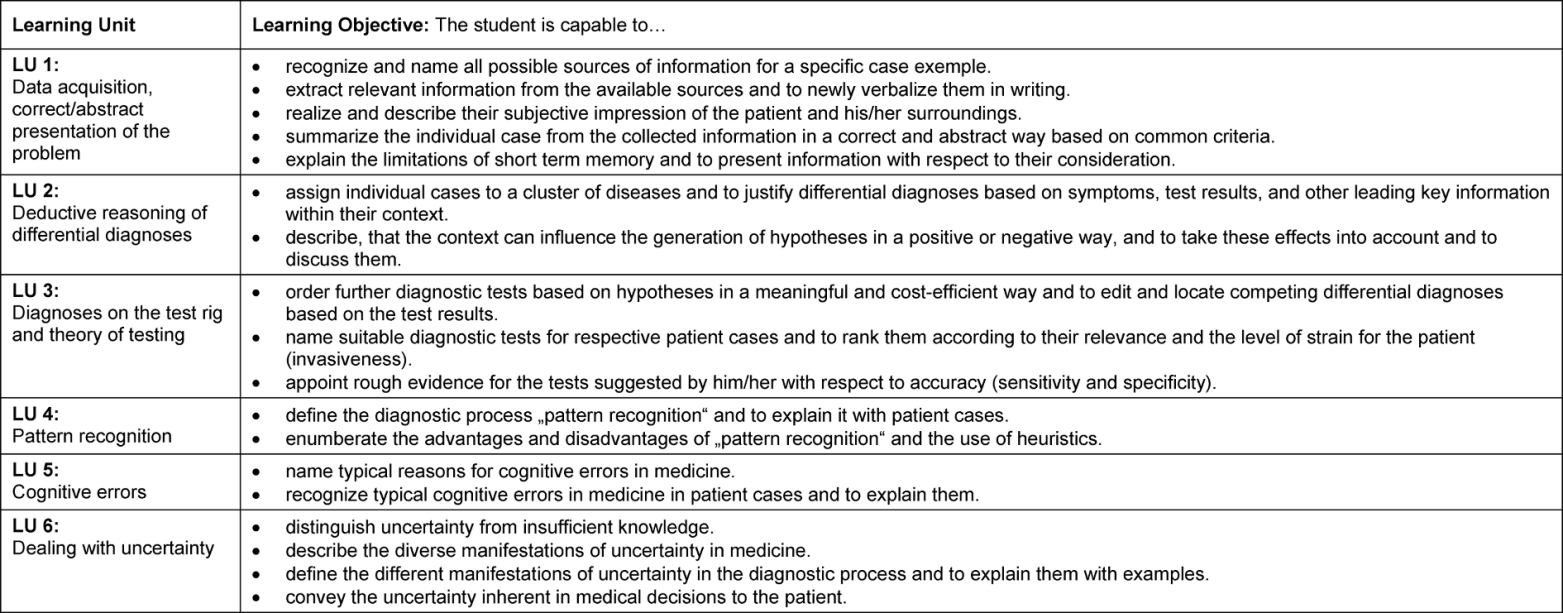
Learning units (LU) and Learning Objectives of the course „Clinical Reasoning“

**Table 2:**
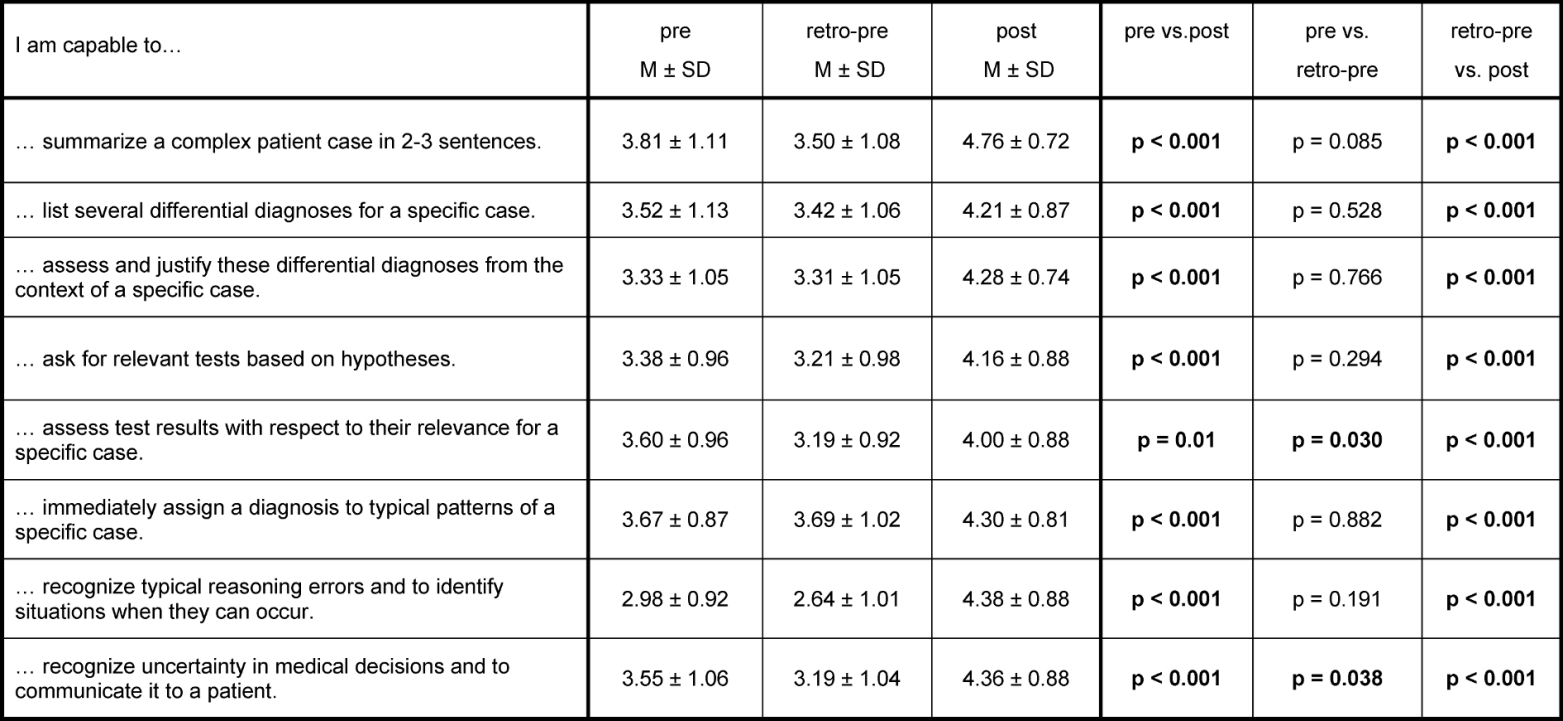
Students’ self-assessment regarding their clinical reasoning skills

**Table 3:**
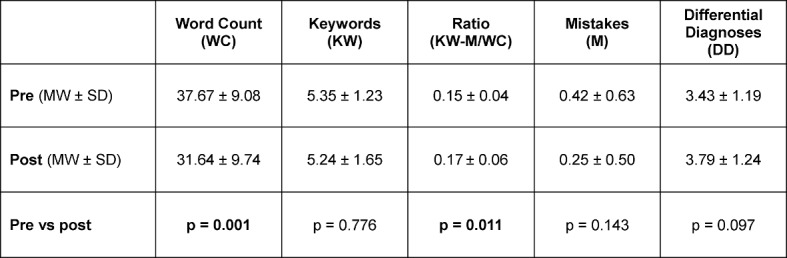
Working with the paper case
